# Virtual Interactive Surgical Skills Classroom: Protocol for a Parallel-Group, Noninferiority, Adjudicator-Blinded, Randomized Controlled Trial (VIRTUAL)

**DOI:** 10.2196/28671

**Published:** 2021-07-22

**Authors:** Arjun Nathan, Monty Fricker, Sonam Patel, Maria Georgi, Man Kien Hang, Aqua Asif, Amil Sinha, William Mullins, Jessie Shea, Nancy Hanna, Benjamin Lamb, John Kelly, Ashwin Sridhar, Justin Collins

**Affiliations:** 1 University College London London United Kingdom; 2 Newcastle University Newcastle United Kingdom; 3 University of Cambridge Cambridge United Kingdom; 4 Cambridge University Hospitals NHS Foundation Trust Cambridge United Kingdom

**Keywords:** digital education, digital health, education, surgery, surgical skills, surgical training, surgical, suturing, telemedicine, virtual classroom, virtual training

## Abstract

**Background:**

Traditional face-to-face training (FFT) for basic surgical skills is inaccessible and resource-intensive. Noninteractive computer-based learning is more economical but less educationally beneficial. Virtual classroom training (VCT) is a novel method that permits distanced interactive expert instruction. VCT may optimize resources and increase accessibility.

**Objective:**

We aim to investigate whether VCT is superior to computer-based learning and noninferior to FFT in improving proficiency in basic surgical skills.

**Methods:**

This is a protocol for a parallel-group, noninferiority, randomized controlled trial. A sample of 72 undergraduates will be recruited from 5 medical schools in London. Participants will be stratified by subjective and objective suturing experience level and allocated to 3 intervention groups at a 1:1:1 ratio. VCT will be delivered using the BARCO weConnect software, and FFT will be provided by expert instructors. Optimal student-to-teacher ratios of 12:1 for VCT and 4:1 for FFT will be maintained. The assessed task will be interrupted suturing with hand-tied knots.

**Results:**

The primary outcome will be the postintervention Objective Structured Assessment of Technical Skills score, adjudicated by 2 experts blinded to the study and adjusted for baseline proficiency. The noninferiority margin (*δ*) will be defined using historical data.

**Conclusions:**

This study will serve as a comprehensive appraisal of the suitability of virtual basic surgical skills classroom training as an alternative to FFT. Our findings will assist the development and implementation of further resource-efficient, accessible, virtual basic surgical skills training programs during the COVID-19 pandemic and in the future.

**Trial Registration:**

International Standard Randomized Controlled Trial Number ISRCTN12448098; https://www.isrctn.com/ISRCTN12448098

**International Registered Report Identifier (IRRID):**

PRR1-10.2196/28671

## Introduction

### Background and Rationale

Basic surgical skills are essential to a wide range of medical professionals and aspiring surgeons. Recent advances in technology have facilitated the development of novel virtual training methods. Virtual classroom training (VCT) permits socially distanced interactive expert instruction via live video communication supplemented by concurrent feedback, graphical aids, and polling [1]. We aim to validate VCT as a modality of high-quality surgical skills education by evaluating its efficacy relative to that of the current alternatives.

### Choice of Comparators

The General Medical Council stipulates safe basic wound closure under supervision as a procedural skill–learning outcome for undergraduate medical students [2]. Despite this, wound closure has not been sufficiently incorporated in university curricula, resulting in a deficiency in graduate competence in recent years [3,4]. Student-led surgical societies can deliver extracurricular tutorials on surgical skills; however, accredited, high-quality training can only be attained by professionally run face-to-face training (FFT) courses [5]. FFT is logistically complex, inaccessible, and resource-intensive. Furthermore, the requirement for social distancing during the COVID-19 pandemic has led to the suspension of nonessential FFT programs [6]. Noninteractive computer-based learning (CBL) is a cost-effective alternative. It can be completed independently by students, with the sole requisite of access to equipment and prerecorded training material. CBL is cheaper and more accessible than FFT; however, it does not offer live demonstration, interaction, or feedback. Therefore, it may be less educationally beneficial [7]. VCT may optimize resources and increase training accessibility while retaining the quality of FFT. VCT has the potential to improve the global availability and accessibility of basic surgical skills training; however, its efficacy has not yet been firmly established [8,9].

### Objectives

This trial is designed to assess the effect size of the 3 interventions and test both the superiority of VCT to CBL and its noninferiority to FFT. We hypothesize that the efficacy of VCT will be greater than that of CBL and similar to that of FFT. We also aim to assess and compare the feasibility and accessibility of the 3 training modalities.

### Trial Design

This is a parallel group, adjudicator-blinded, randomized controlled trial. Participants will be divided into 3 groups with an allocation ratio of 1:1:1. Group 1 will undertake noninteractive CBL, group 2 will receive VCT, and group 3 will receive FFT.

## Methods

### Participants, Interventions, and Outcomes

#### Study Setting

Participants will be recruited from across all year-groups within 5 medical schools in London, United Kingdom, to reduce travel requirements during the COVID-19 pandemic. The participating universities include the following: University College London (UCL), Imperial College London, King’s College London, St George’s University of London, and Barts and the London School of Medicine and Dentistry. The opportunity for students to participate will be advertised on the internet by the official student-led surgical societies that represent the 5 medical schools. Eligible individuals will be invited to volunteer through an electronic application form, which will contain a participant information leaflet (PIL), which includes employed methods of data handling and anonymization. The form will request the provision of informed consent and personal data that comprise name, age, sex, university, year of study, and email address for correspondence. The PIL and consent form are attached in Multimedia Appendix 1.

#### Eligibility Criteria

The criteria for inclusion will be current medical student status, availability on specified trial dates, and the ability to access both a PC with an integrated camera and a smartphone with an integrated camera. Outcome adjudicators will have membership of the Royal College of Surgeons degrees and Basic Surgical Skills certification [10].

#### Interventions

Expenses associated with the coordination of the interventions are listed in Table 1. All participants will be provided with a suture pad that contains a 5-cm incision, needle holder, surgical scissors, toothed and nontoothed forceps, a 70-cm-long nylon cord, and 5 silk suture packs. Interventions will have a 2-hour duration. All participants will view a 6-minute noninteractive instructional video that demonstrates the correct interrupted suturing and hand-tying technique with annotations before beginning the intervention.

Individuals in the CBL group will participate in the study from a location of their choice. They will practice independently under self-direction with continued access to the instructional video. They will be continuously monitored by a trial coordinator via the videoconferencing platform Zoom [11]. The VCT group will remotely attend a virtual classroom led by 2 expert instructors using the BARCO weConnect platform [1]. The VCT control room will be located at UCL, and students will log in from a location of their choice from a PC. Participants will be trained with an instructor-to-student ratio of 1:12. A proficiency-based progression model will be followed. Instructors will have a live view of all participants and will utilize graphical instructional aids, annotations, and interactive polling to deconstruct the task and define its key maneuvers. Students will display their own hands while practicing, allowing the instructors to provide them with concurrent feedback and guidance. The FFT group will physically attend a socially distanced classroom at UCL, in line with national government and institutional COVID-19 guidelines. The group will be divided into 6 subgroups (n=4 students each), and each subgroup will be trained by a single instructor and will have an instructor-to-student ratio of 1:4. This ratio has been shown to optimize the educational benefit of FFT [12]. A proficiency-based progression model will again be followed, and instructors will perform a live demonstration of the correct technique and verbally deconstruct the procedure and define its key maneuvers. Students will also receive concurrent feedback and guidance from the instructor while they practice. The same instructors will deliver all 3 interventions; they will provide the video for CBL and implement the training for both the VCT and FFT arms.

**Table 1:** Financial costs associated with the coordination of the intervention.

Item or service	Cost (GB £)
Subscription to the Barco WeConnect platform	186.24
In-person training venue fees	510.00
Suturing equipment	988.41
Personal protective equipment	113.09
Zoom videoconferencing plan	11.99
Total cost	1809.73

#### Outcomes

Baseline characteristics will be collected to facilitate the identification of systematic differences between intervention groups that may increase the risk of bias. Age, year group, sex, and hand dominance will be elicited through a preintervention questionnaire. The primary outcome will be postintervention suturing and hand-tying proficiency, adjusted for preintervention proficiency. After the initial instructional video and again immediately after the intervention, participants will complete an assessment task. The task will include placing 3 interrupted sutures with hand-tied knots by using a single silk suture, with a time limit of 4 minutes. Proficiency will be measured using a validated Objective Assessment of Surgical and Technical Skills (OSAT) used by the Royal College of Surgeons [10]. Scores will be adjudicated by 2 experts blinded to the study design. Proficiency will be defined as the mean value of both adjudicator scores.

Secondary outcomes will be the students’ subjective suturing and knot-tying confidence, perceptions of the intervention, and expenses associated with session attendance. Subjective confidence and perceptions will be elicited through a postintervention questionnaire and measured on a 5-point Likert scale. Expenses will be self-reported by participants.

#### Participant Timeline

Recruitment will begin in November 2020. Randomization and allocation will commence in January 2021. Interventions, data collection, and data analysis will be completed by May 2021, depending on governmental and institutional COVID-19 guidelines.

#### Sample Size

The effect of VCT on OSAT-measured proficiency is not reported in the existing literature. The VCT technology implemented by this trial has been optimized for 24 attendees. A total sample size of 72 individuals (n=24 per intervention group) was therefore selected. A sensitivity power analysis was performed using a 2-tailed dependent samples *t* test. With an *α* and *β* error probability of .05, a minimum detectable effect of 0.769 was computed.

#### Recruitment

The application window will be open for 31 days after advertisement. Applicants will be informed of the outcome within 1 week after its closure. From the pool of applicants, a sample of 72 will be selected to participate in the trial. To ensure substantial representation from all 5 centers, 8 participants will be selected at random from each of the medical schools. Further, 32 participants will then be selected randomly from the remaining applicant pool.

### Assignment of Interventions

#### Allocation

A web-based questionnaire will be distributed to the participants to elicit their subjective confidence in their own suturing ability and the quantity of formal (expert-led) and informal (peer-led or independent) suturing training they have previously received. Participants will then be stratified by subjective suturing confidence—measured on a 5-point Likert scale—and objective previous suturing training experience—measured to the nearest hour. Permuted block randomization (block size=3) will be carried out to allocate the participants within each stratum to 1 of the 3 intervention groups. Permutations will be selected by a random number generator. Comprehensive instructions and guidelines to be followed before and during the training sessions will also be provided. Participants who do not want or are unable to participate will be replaced through random selection from the remaining list of applicants where possible.

#### Blinding

Outcome adjudicators alone will be blinded to intervention assignment. When recording the assessment task video, desk surfaces will be covered with plain white paper, and participants will not be visible above the elbow. Video files will be renamed and reordered through random number generation prior to adjudicator review.

### Data Collection, Management, and Analysis

#### Data Collection

Applicants will complete a questionnaire that elicits personal data, which will be used for correspondence. Participants will complete a questionnaire immediately before and after the intervention. All questionnaires will be administered using Jisc Online Surveys [13]. Participants will record the assessment tasks by using a personal device with an integrated camera. Framing specifications will be provided in advance. Video files will be shared with trial coordinators securely via WeTransfer [14].

#### Data Management

All data will be stored on encrypted hard-drives under password protection. Personal data collected from applicants will be accessed by trial coordinators only to permit the collection of nonidentifiable information from participants. Participants will be assigned anonymous numeric IDs, which will be used to identify their video recordings and questionnaire responses.

#### Statistical Methods

Within-group proficiency improvements after the interventions will be analyzed using the dependent *t* test. Between-group postintervention proficiency will be assessed through analysis of covariance with baseline proficiency as a covariate in the regression model. Post hoc analysis will be performed using the Fisher test of least significant difference. The combined hypothesis that VCT is both superior to CBL and noninferior to FFT will be tested at a 95% significance level. The noninferiority margin (*δ*) was defined on the basis of historical data. An observational study investigated the effect of FFT on OSAT scores, with a reported difference of 3.7 (95% CI 2.7-4.7) in mean values [15]. A *δ* of 0.675 was selected to ensure a preserved fraction of 3/4.

### Monitoring

#### Data Monitoring

There will be no data monitoring as data collection will not be continuous; instead, all data will be collected simultaneously, with no interim analysis.

#### Harms and Auditing

Harms and auditing will be performed with ethical approval and participant consent form (Multimedia Appendix 1).

### Ethics and Dissemination

#### Research Ethics Approval

The study has received ethical approval from the UCL Research Ethics Committee (REC) (ID: 19071/001). National and institutional COVID-19 guidelines will be followed and have been discussed and agreed upon with the UCL REC.

#### Protocol Amendments

Amendments to the protocol will be submitted to the UCL REC for consideration. Deviations in the statistical plan will be described and justified in updated versions of the protocol.

#### Consent

Informed consent will be obtained using the PIL and consent form, which are provided in Multimedia Appendix 1.

#### Confidentiality

Identifiable data will be collected from participants solely for the purposes of correspondence. They will be stored separately from nonidentifiable data and destroyed on completion of the study.

#### Data Access

The final anonymized data set will be accessed by the statistician alone. All other data will be destroyed on study completion.

#### Ancillary and Posttrial care

In accordance with the consent form, harms are not expected and risks are expected to be low, and data can be withdrawn by the participant up until the point of data analysis.

#### Dissemination Policy

The findings of this trial will be submitted for local, national, and international presentation and publication in a peer-reviewed journal. Authorship will be in accordance with the ICMJE (International Committee of Medical Journal Editors) guidelines. The anonymized participant data set and statistical code will be made available to the journal for peer-review on request.

## Results

The study was fully funded in December 2020 and approved by the UCL REC in December 2020. The study is currently underway, and data collection has started. Data analysis and publication of results are expected in August 2021.

## Discussion

### Expected Findings

This randomized trial will be a comprehensive analysis of the suitability of virtual basic surgical skills classroom training as an alternative to FFT and CBL. Our findings will allow for the development and implementation of further resource-efficient, accessible virtual training programs during the COVID-19 pandemic and in the future.

### Strengths and Limitations

We plan to conduct a 3-arm randomized controlled trial that will investigate the effect of VCT. Comparator groups will be noninteractive CBL and FFT. We will use a validated OSAT score to assess proficiency. We shall use optimal teaching methods and follow COVID-19 guidelines.

## Appendix

Multimedia Appendix 1 Participation information leaflet and participant consent form.

## Abbreviations

CBL: computer-based learning
FFT: face-to-face training
OSAT: Objective Structured Assessment of Technical Skills
PIL: participant Informal leaflet
REC: Research Ethics Committee
UCL: University College London
VCT: virtual classroom training
