# Supplementary material for: Virtual Interactive Surgical Skills Classroom: Protocol for a Parallel-Group, Noninferiority, Adjudicator-Blinded, Randomized Controlled Trial (VIRTUAL)
Source: JMIR Res Protoc. 2021 Jul 22;10(7):e28671. doi: 10.2196/28671 (PMC8367109; doi:10.2196/28671)
Supplement: Multimedia Appendix 1 [file resprot_v10i7e28671_app1.docx]

**Multimedia Appendix 1.** Participation information leaflet and participant consent form.

**1. Participant Informal Leaflet (PIL)**

1. Invitation

We invite you to take part in a research study. In this study, we are investigating the use of virtual interactive teaching as a method to teach technical surgical skills compared to non-interactive video teaching and face-to-face interactive teaching.

2. What is the project’s purpose?

Our aim is to study the impact of novel virtual teaching methods in improving technical surgical skills. We intend to compare three different modalities of practical surgical skills teaching: limited non-interactive video teaching, face-to-face teaching and virtually interactive teaching. Furthermore, we aim to assess the accessibility, cost and convenience of virtual teaching compared to other teaching modalities. We have an ultimate aim to improve medical education and knowledge from the research we carry out.

3. Why have I been chosen?

We hope to better understand the role of virtual teaching methods in the undergraduate teaching of surgical skills. We are choosing medical students from all year groups across all medical schools in the UK.

Inclusion criteria includes:

- Registered medical student at London medical school

- London medical schools are limited to University College London, Imperial College London, St. Georges, Queen Mary’s and Kings College London.

4. Do I have to take part?

Your participation in this study is completely voluntary. If you do decide to take part, you will be given this information sheet to keep (and be asked to sign a consent form). You can withdraw at any time without giving a reason and without it affecting any benefits that you are entitled to. If you decide to withdraw you will be asked what you wish to happen to the data you have provided up to that point and you have the option to permanently delete the storage of your data. You can withdraw your data up to the point of data analysis, at which point your data will be incorporated into the study permanently. Please contact us below if you would like to withdraw your data and we will support you with this if possible.

5. What will happen to me if I take part?

First, we will give you a questionnaire to complete based on your confidence in completing a certain surgical task and you will also undertake this surgical task. You will then be randomly assigned to a group receiving one of the three teaching methods (non-interactive video viewing, face-to-face interactive teaching or virtual interactive teaching). Having received teaching, you will then undertake the surgical skill again and complete a post-teaching questionnaire. You will have supervisors available to you during the process if there are any concerns or questions. You may stop the process at any time or for any reason if you so wish. You need to be available for participation in this study from the 14th November to 14th March. You may be required to participate in scheduled virtual classroom teaching at a location of your convenience or face-to-face teaching in London.

6. Will I be recorded and how will the recorded media be used?

The video recordings of your activities made during this research will only be used for analysis and for illustration in conference presentations and lectures. All recorded videos will be pseudo-anonymised. The field of view of the camera will be set so it only captures the hands of the participant and the equipment. No identifiable images will be recorded. Images will be saved onto UCL OneDrive under your participant ID. No other use will be made of them without your written permission, and no one outside the project will be allowed access to the original recordings. The questionnaires you fill out will remain anonymous.

7. Will my taking part in this project be kept confidential?

All the information that we collect about you during the course of the research will be kept strictly confidential. You will not be able to be identified in any ensuing reports or publications. All answers will be anonymised, the data will be collected and handled in an anonymised and protected way. Participants have the right to withdraw from the study at any time or withdraw their data at any time up to the point that data analysis commences. All data captured as part of this study will be secured in encrypted password protected drives only accessible to the research team.

8. Limits to confidentiality

Please note that assurances on confidentiality will be strictly adhered to unless evidence of wrongdoing or potential harm is uncovered. In such cases the University may be obliged to contact relevant statutory bodies/agencies.

9. What will happen to the results of the research project?

The results from this research project will be analysed and the data may be published or used as the basis for further research. The results may be used as a basis for further research. Participants will be offered the opportunity to view the results as a presentation that will be distributed to participants on request.

10. Local Data Protection Privacy Notice

Notice: The controller for this project will be University College London (UCL). The UCL Data Protection Officer provides oversight of UCL activities involving the processing of personal data, and can be contacted at data-protection@ucl.ac.uk

This ‘local’ privacy notice sets out the information that applies to this particular study. Further information on how UCL uses participant information can be found in our ‘general’ privacy notice:

For participants in health and care research studies, click here

The information that is required to be provided to participants under data protection legislation (GDPR and DPA 2018) is provided across both the ‘local’ and ‘general’ privacy notices.

The categories of personal data used will be as follows:

• Name

• Email address

• Age

• Sex

• Medical school

• Year of study

The lawful basis that would be used to process your personal data will be performance of a task in the public interest. The lawful basis used to process special category personal data will be for scientific and historical research or statistical purposes.

Your personal data will be processed so long as it is required for the research project. we will anonymise the personal data you provide we will undertake this, and will endeavour to minimise the processing of personal data wherever possible.

If you are concerned about how your personal data is being processed, or if you would like to contact us about your rights, please contact UCL in the first instance at data-protection@ucl.ac.uk.

11. Who is organising and funding the research?

This project has been kindly supported by University College London (UCL) and the Royal College of Surgeons England (RCS).

12. Contact for further information

Please contact Sonam Patel (sonam.patel.16@ucl.ac.uk) for further information

13. Concerns or Complaints

If you do have any concerns or wish to register a complaint please contact the principal investigator Arjun Nathan (arjun.nathan.11@ucl.ac.uk). If the response is lacking or not satisfactory please contact UCL Research Ethics Committee (ethics@ucl.ac.uk).

Thank you for reading this information sheet and for considering taking part in this research study

**2. Participant Consent Form**

Thank you for considering taking part in this research. The person organising the research must explain the project to you before you agree to take part. If you have any questions arising from the Information Sheet or explanation already given to you, please ask the researcher before you decide whether to join in. You will be given a copy of this Consent Form to keep and refer to at any time.

• *I confirm that I have read and understood the Information Sheet for the above study. I have had an opportunity to consider the information and what will be expected of me. I have also had the opportunity to ask questions which have been answered to my satisfaction

• *I understand that I will be able to withdraw my data up to 2 weeks after completion of questionnaires and filming. After a period of 2 weeks, data analysis will commence, and data may not be withdrawn.

• *I voluntarily consent to participate in the study.

• *I understand that all personal information will remain confidential and that all efforts will be made to ensure I cannot be identified. Use of information for this project only: Data processed for research purposes will be stored under password protection and accessed only by researchers involved in the study.

• I understand that my data gathered in this study will be stored anonymously and securely. It will not be possible to identify me in any publications.

• *I understand that my information may be subject to review by responsible individuals from the University College London (UCL) for monitoring and audit purposes.

• I understand that my participation is voluntary and that I am free to withdraw at any time without giving a reason, without the care I receive or my legal rights being affected. I understand that if I decide to withdraw, any personal data I have provided up to that point will be deleted unless I agree otherwise.

• I understand there is no promise or guarantee of benefits which have been made to encourage me to participate.

• I understand that the data will not be made available to any commercial organisations but is solely the responsibility of the researcher(s) undertaking this study.

• I understand that I will not benefit financially from this study or from any possible outcome it may result in in the future.

• I agree that my anonymised research data may be used by others for future research. [No one will be able to identify you when this data is shared.]

• I understand that the information I have submitted will be published as a report and I wish to receive a copy of it.

• I consent to my suturing being video recorded and understand that the recordings will be stored anonymously, using password-protected software and will be used for training, quality control, audit and specific research purposes.

• I hereby confirm that I understand the inclusion criteria as detailed in the Information Sheet explained to me by the researcher.

• I am aware of who I should contact if I wish to lodge a complaint.

• I understand that other authenticated researchers will have access to my anonymised data.

• I understand that by not giving consent I may be deemed ineligible for the study.

I confirm that I have read, understood and agreed to all of the points listed above. I understand that I am consenting to all elements of the study.

Signed__________________
